# Antipsychotic prescribing patterns in a state psychiatric hospital with predominantly forensic admissions

**DOI:** 10.3389/fpsyt.2025.1736853

**Published:** 2026-01-30

**Authors:** Nina Vadiei

**Affiliations:** 1 College of Pharmacy, The University of Texas at Austin, Austin, TX, United States; 2 UT Health San Antonio, San Antonio, TX, United States; 3 San Antonio State Hospital, San Antonio, TX, United States

**Keywords:** antipsychotic, forensic-psychiatric practice, pattern, prescribing, state hospital

## Abstract

**Background:**

There are currently no published studies evaluating antipsychotic prescribing patterns in state hospital settings in the United States. This data is important to review since state hospitals involuntarily commit patients with serious mental illness (SMI) to receive treatment with antipsychotic medications, which are associated with a multitude of dose-dependent adverse effects.

**Methods:**

This cross-sectional study describes antipsychotic prescribing patterns at a single state psychiatric hospital with predominantly forensic admissions. Data was collected via chart review at a single point in time, including demographic data and clinical characteristics such as admission type, neuropsychiatric diagnoses, length of stay, antipsychotic selection and dosing, and total number of scheduled antipsychotics. If the total daily dose of the scheduled antipsychotic was greater than the recommended typical maximum dose, it was considered ‘high-dose’ antipsychotic use.

**Results:**

Among 212 state hospital admissions, nearly two-thirds of patients were prescribed ≥ 1 antipsychotic at either the recommended typical maximum dose (N=76; 35.8%) or above the typical maximum dose (N=62; 29.2%). Several patients were prescribed antipsychotic polypharmacy (APP) (two scheduled antipsychotics [N = 76; 35.8%]; three scheduled antipsychotics [N = 13; 6.1%]). About one-third of patients were prescribed olanzapine (N = 76; 35.8%), and about a quarter of patients were prescribed clozapine (N = 51; 24.1%) and/or paliperidone (including Invega Sustenna) (N = 49; 23.1%). One-fifth of patients were prescribed a first-generation antipsychotic (N = 43; 20.3%). Nearly one-third of patients were prescribed a long-acting injectable antipsychotic (LAIA) (N = 69; 32.5%).

**Conclusion:**

High-dose antipsychotic use and APP was common in this U.S. state psychiatric hospital. Larger, multicenter studies are needed to determine how antipsychotic prescribing patterns vary between U.S. state hospitals. Testing the development and implementation of antipsychotic stewardship programs in state hospital settings is recommended to establish best practices for monitoring antipsychotic use patterns and associated health outcomes.

## 1 Introduction

Patients with untreated serious mental illness (SMI) with forensic involvement are typically admitted to the state psychiatric hospital for treatment. State psychiatric hospitals play a crucial role in treating patients with SMI, including uninsured and indigent patients; pretrial defendants or jail inmates being “restored” to competency to stand trial; criminal defendants found “unrestorable” who remain hospitalized under civil commitment criteria; and individuals who are considered a danger to themselves or others secondary to a mental illness (1). Over time, state psychiatric hospitals have had a substantial rise in forensic referrals (2). Patients with SMI and forensic involvement are highly vulnerable (3), as they are involuntarily committed to receive treatment. Antipsychotics (the recommended first-line treatment for psychotic illnesses) pose a risk of numerous dose-dependent adverse effects (4), and as such, it is essential to establish high-quality standardized processes for monitoring health outcomes associated with antipsychotic use in patients on involuntarily commitments.

International studies report that high-dose antipsychotic use and antipsychotic polypharmacy (APP) are common in forensic psychiatric settings, despite limited evidence to support such practices (5–9). There are currently no studies that describe antipsychotic prescribing patterns in state psychiatric hospitals in the United States (U.S.). Forensic settings, such as state psychiatric hospitals, are particularly important to investigate antipsychotic prescribing patterns and associated health outcomes in, as they have been referred to by psychiatric clinician experts as “the last resort of the mental health system (1).” Forensically involved patients possess unique factors that may contribute to non-evidence-based antipsychotic prescribing practices (10). For example, they are more likely to have a history of engaging in aggressive/violent behavior (10), which may subsequently lead to more ‘aggressive’ antipsychotic treatment strategies, such as utilizing higher doses or APP. Patients with treatment refractory illness have often failed multiple standard medication regimens, further contributing to non-evidence based antipsychotic prescribing practices. Length of stays tend to be longer for forensic admissions, which may also contribute to APP and greater dose titrations in patients who continue to experience intermittent episodes of aggression. Despite these unique treatment challenges, a systematic review published in 2020 highlights the shortage of knowledge on the effectiveness of pharmacologic treatment within forensic psychiatry, calling for high-quality studies in this setting (10). While guidelines for forensic psychiatric treatment have been proposed, the evidence base for forensic-psychiatric practice is weak (11).

There are many barriers to researching health outcomes associated with antipsychotic use in forensic settings (3, 12). Optimal antipsychotic dosing is essential for maximizing therapeutic effectiveness, ensuring patient safety, and improving treatment adherence. Yet, the subjective nature of assessing treatment response in psychiatry contributes to variable dosing strategies (13), with higher doses and APP often justified on the grounds of the patient having a higher severity of illness (14). For the many patients who don’t respond to higher doses or APP, gradual deprescribing interventions are recommended to improve tolerability and reduce the risk of future adverse effects (15). However, evidence suggests that patients on high-dose antipsychotics/APP are often continued on the same regimen until transfer/discharge (7).

The lack of high-quality clinical research and treatment guidelines for forensically involved patients with SMI makes it challenging to establish and enforce best practices. However, this is an issue in dire need of addressing, considering the demand for forensic psychiatric treatment continues to grow (2, 16, 17), and restrictions to recruiting patients for clinical research in this area will likely remain (3). Key community partners and policymakers will need to invest in novel interventions for feasible and reliable monitoring/reporting of health outcomes for patients prescribed antipsychotics in state psychiatric hospitals (18). Development and implementation of ‘Psychotropic Stewardship Programs’ (PSPs) (19), or ‘Antipsychotic Stewardship Programs’ (ASPs) (20), have been proposed as a strategy for optimizing the safe and effective use of psychotropics in various treatment settings. However, various barriers to implementation of PSPs/ASPs are noted, such as administrative support and additional resources needed to make the service sustainable. As such, reporting data to support the need for ASP development/implementation to community partners and policymakers is crucial. This study aims to (1) describe antipsychotic prescribing patterns at a large state psychiatric hospital in the U.S., and (2) provide rationale for prioritizing the development and implementations of ASPs in state psychiatric hospital systems.

## 2 Materials and methods

### 2.1 Study design and data source

This cross-sectional study used chart data from one state psychiatric hospital. All patient charts for active admissions were evaluated at a single point in time in September 2025. The University of Texas Health San Antonio Institutional Review Board approved the study protocol prior to evaluation of patient data.

### 2.2 Study population

Most patients at San Antonio State Hospital (SASH) are considered ‘forensic’ admissions, meaning they have either been charged with a crime but necessitate mental health treatment to restore competency to stand trial, or have been convicted as ‘Not Guilty by Reason of Insanity (NGRI),’ but require further treatment before being considered safe for community reintegration. These patients tend to have a higher severity of illness and a long history of episodic relapses. While most admissions to SASH are involuntary commitments, there were a few ‘voluntary’ status admissions at the time of chart review. Voluntary admissions at SASH typically face distinct barriers to transitioning to alternative, less-secure treatment settings. All active admissions (involuntary and voluntary, forensic and non-forensic) were included and categorized to compare differences in antipsychotic prescribing between all admission types. SASH is a smoke-free facility and has a tobacco-free policy, meaning smoking is prohibited on all hospital grounds, including buildings and parking lots. The policy applies to all patients, visitors, and staff, and there are no designated outdoor spaces for smoking.

### 2.3 Study variables and definitions

The following data were collected via chart review: age, sex, race/ethnicity, admission type ([1] forensic commitment, competency restoration; [2] forensic commitment, NGRI acquittal; [3] civil commitment; [4] voluntary), neuropsychiatric diagnoses, length of stay, number of scheduled antipsychotics, antipsychotic dose (dose below recommended typical range [yes/no], dose in recommended typical range [yes/no]; dose above the typical maximum [yes/no]; dose at typical maximum [yes/no]), antipsychotic prescribed, adjunct psychotropics prescribed (e.g., mood stabilizer, antidepressant, benzodiazepine), and total number of scheduled psychotropic medications (defined as any medication being used to treat a neuropsychiatric indication/symptom per the charted indication). Mood stabilizer was defined as lithium or an antiepileptic mood stabilizer used to target neuropsychiatric symptoms. Typical dose ranges were based on the American Psychiatric Association (APA) Practice Guideline for the Treatment of Patients with Schizophrenia (4). If the total daily dose of the antipsychotic prescribed was greater than the recommended typical maximum dose it was considered ‘high-dose’ antipsychotic use. For first-generation antipsychotics, the end of the ‘typical dose range’ was used as the ‘typical maximum dose’ rather than the listed ‘maximum daily dose,’ since the maximum doses for first-generation antipsychotics are far greater than the recommended typical dose range (see Table 1 of the APA Practice Guideline for the Treatment of Patients with Schizophrenia) (4). If a patient was prescribed both the oral and long-acting injectable (LAI) formulation of the same antipsychotic outside of the recommended window of using oral supplementation when initiating LAIs, then each order was counted as a separate antipsychotic (APP). LAI doses were converted to the equivalent oral dose when determining the total daily dose of each antipsychotic.

### 2.4 Statistical analysis

Descriptive statistics were used for demographic and clinical characteristics.

## 3 Results

A total of 212 patient charts were reviewed for data collection and analysis. Patient demographics and clinical characteristics are provided in **Table 1**. Most patients were male (N = 158; 74.5%), were admitted on a forensic commitment for competency restoration (N = 147; 69.3%), and had a primary diagnosis of schizophrenia (N = 96; 45.3%) or schizoaffective disorder (N = 100; 47.2%). Approximately half of the patients were Hispanic (N = 107; 50.5%). The median length of stay was 2.3 years (IQR 0.3 to 2.9).

**Table 1:** Demographics and clinical characteristics (N = 212).

Characteristic	Value
Age, mean (SD)	44.1 (13.8)
Male sex, n (%)	158 (74.5)
Race/Ethnicity, n (%)	
White	61 (28.8)
Black	41 (19.3)
Hispanic	107 (50.5)
Asian	3 (1.4)
Admission type, n (%)	
Forensic commitment, competency restoration	147 (69.3)
Forensic commitment, NGRI	42 (19.3)
Civil commitment	21 (9.9)
Voluntary	2 (0.9)
Diagnoses, n (%)^a^	
Schizophrenia	96 (45.3)
Schizoaffective disorder	100 (47.2)
Mood disorder	14 (6.6)
Substance use disorder	81 (38.2)
Anxiety disorder	11 (5.2)
Personality disorder	20 (9.4)
Traumatic brain injury	8 (3.8)
Neurocognitive disorder	20 (9.4)
Seizure disorder	10 (4.7)
Developmental disorder	30 (14.2)
Length of Stay, years, median (IQR)	2.3 (0.3, 2.9)

SD, standard deviation; NGRI, not guilty by reason of insanity; IQR, interquartile range

a One or more diagnoses included per patient.

Several patients were prescribed APP (two scheduled antipsychotics [N = 76; 35.8%]; three scheduled antipsychotics [N = 13; 6.1%]). About one-third of patients were prescribed olanzapine (N = 76; 35.8%), and about a quarter of patients were prescribed clozapine (N = 51; 24.1%) and/or paliperidone (including Invega Sustenna) (N = 49; 23.1%). One-fifth of patients were prescribed a first-generation antipsychotic (N = 43; 20.3%), and a similar proportion of patients were prescribed a scheduled benzodiazepine (N = 42; 19.8%). Approximately half of patients were prescribed an adjunct mood stabilizer (N = 109; 51.4%). Nearly one-third of patients were prescribed a long-acting injectable antipsychotic (LAIA) (N = 69; 32.5%). Patients were prescribed an average of three scheduled psychotropic medications.

Nearly two-thirds of patients were prescribed antipsychotics at either the recommended typical maximum dose (N = 76; 35.8%) or above the typical maximum dose (N = 62; 29.2%) (**Table 2**). The most commonly used antipsychotics were often prescribed at the typical maximum dose or above it. For example, olanzapine was often dosed at daily doses of greater than 20 milligrams (mg) per day (median 30 mg; IQR 20–40 mg). On the other hand, clozapine was often dosed on the lower end of the recommended typical dose range (median 300 mg; IQR 212.5–475 mg). High-potency first-generation antipsychotics, such as haloperidol and fluphenazine, were often dosed above the recommended typical maximum dose (haloperidol median 20 mg, IQR 10–30 mg; fluphenazine median 25 mg, IQR 20–35 mg). Additional antipsychotic dosing patterns from the study sample are outlined in **Table 3**.

**Table 2:** Psychotropic prescribing patterns (N = 212).

Patient information	Value
One scheduled antipsychotic, n (%)	123 (58.0)
Two scheduled antipsychotics, n (%)	76 (35.8)
Three scheduled antipsychotics, n (%)	13 (6.1)
≥ 1 antipsychotic dosed below recommended typical range, n (%)	9 (4.3)
≥ 1 antipsychotic dosed in recommended typical range, n (%)	65 (30.7)
≥ 1 antipsychotic dosed at recommended typical maximum, n (%)	76 (35.8)
≥ 1 antipsychotic dosed above recommended typical maximum, n (%)	62 (29.2)
Antipsychotic prescribed, n (%)	
Haloperidol^a^	22 (10.4)
Oral haloperidol	19 (9.0)
Haloperidol decanoate LAI	4 (1.9)
Fluphenazine^a^	15 (7.1)
Oral fluphenazine	12 (5.7)
Fluphenazine decanoate LAI	4 (1.9)
Chlorpromazine	3 (1.4)
Loxapine	2 (0.9)
Perphenazine	1 (0.5)
Aripiprazole	28 (13.2)
Oral aripiprazole	18 (8.5)
Aripiprazole monohydrate LAI	10 (4.7)
Asenapine	1 (0.5)
Cariprazine	2 (0.9)
Clozapine	51 (24.1)
Lumateperone	1 (0.5)
Lurasidone	4 (1.9)
Olanzapine	76 (35.8)
Quetiapine	28 (13.2)
Paliperidone	49 (23.1)
Oral paliperidone	6 (2.8)
Invega Sustenna LAI	43 (20.3)
Risperidone^a^	26 (12.3)
Oral risperidone	19 (9.0)
Risperidone (Consta) LAI	5 (2.4)
Risperidone (Uzedy) LAI	3 (1.4)
Xanomeline and Trospium	1 (0.5)
Adjunct mood stabilizer prescribed, n (%)	109 (51.4)
Adjunct antidepressant prescribed, n (%)	55 (25.9)
Adjunct benzodiazepine prescribed, n (%)	42 (19.8)
Total number of scheduled psychotropics per patient, mean (SD).	3 (1.3)

LAI: long-acting injectable; SD: standard deviation

a Select patients were prescribed both the oral + LAI formulation outside the recommended oral supplementation period.

**Table 3:** Antipsychotic dosing (N = 310).

Antipsychotic	Dose (mg/day), median (IQR)^b^
Haloperidol (n=22)	20 (10-30)
Fluphenazine (n=15)	25 (20-35)
Chlorpromazine (n=3)	200 (175-225)
Loxapine (n=2)	87.5 (81.25-93.75)
Perphenazine (n=1)	24 (24)
Aripiprazole (n=28)	27.5 (18.8-30)
Asenapine (n=1)	20 (20)
Cariprazine (n=2)	3.75 (3.4-4.1)
Clozapine (n=51)	300 (212.5–475)
Lumateperone (n=1)	42 (42)
Lurasidone (n=4)	100 (64-130)
Olanzapine (n=76)	30 (20-40)
Quetiapine (n=28)	600 (300-800)
Paliperidone (n=49)	12 (9-12)
Risperidone (n=26)	4 (4-6)
Xanomeline and Trospium (n=1)	50/20 (50/20)
Dose below recommended typical range, n (%)^a^	36 (11.5)
Dose in recommended typical range, n (%)^a^	123 (39.4)
Dose at recommended typical maximum, n (%)^a^	82 (27.9)
Dose above recommended typical maximum, n (%)^a^	69 (21.2)

IQR, interquartile range.

Note: long-acting injectable doses were converted to the equivalent daily oral dose. For patients on both oral + LAI formulation outside of the recommended oral supplementation window (N = 3), the daily dose of both were added together to determine the dosing category.

a Typical dose ranges per the American Association American Psychiatric Association Practice Guideline for the Treatment of Patients with Schizophrenia (4): haloperidol (5–20 mg/day); fluphenazine (6–20 mg/day); chlorpromazine (200–800 mg/day); loxapine (60–100 mg/day); perphenazine (8–32 mg/day); aripiprazole (10–30 mg/day); asenapine (20 mg/day); cariprazine (1.5–6 mg/day); lumateperone (42 mg/day); lurasidone (40–160 mg/day); olanzapine (10–20 mg/day); quetiapine (400–800 mg/day); paliperidone (3–12 mg/day); risperidone (2–8 mg/day); xanomeline and trospium (100/20-125/30 mg/day). Typical dose ranges for lumateperone and xanomeline and trospium were obtained from the manufacturer package inserts (42, 43).

b Reported as median (IQR) due to data being nonparametric.

## 4 Discussion

To the best of my knowledge, this is the first U.S. study to describe antipsychotic prescribing patterns in a state psychiatric hospital comprising mostly forensic admissions. Similar to international studies describing antipsychotic prescribing patterns in forensic institutions (5–9), high-dose antipsychotic use and APP were common.

The prevalence of APP appears to vary drastically between forensic institutions, ranging from 22.2% (N = 189) in one single-center, cross-sectional study conducted at a United Kingdom ‘high security hospital (9),’ to 41.9% (N = 212) in the current U.S. study, to 85.8% (n=289) in a study conducted at a Swiss forensic hospital between 1995 and 2016 (7). In previous studies where antipsychotic dosing was reported in addition to APP prevalence, heterogenous methods were used to describe antipsychotic dosing, making it difficult to draw useful comparisons (6–9). For example, in one Canadian cross-sectional study by Farrell et al. (8), to determine if a patient was prescribed a ‘high dose’ of antipsychotic(s), the prescribed dosage was converted to a percentage of the maximum recommended dose using the Canadian version of the Prescribing Observatory for Mental Health (POMH) antipsychotic dosage ready reckoner (21), which defines typical maximum doses differently than that of the APA guidelines referenced in this study (for example, risperidone is listed as having a maximum adult daily dose of 16 mg per day in the POMH, as opposed to 8 mg per day in the APA guidelines). In the study by Farrell et al. (n=142), 54.9% of patients were prescribed APP, and 40.1% were on ‘high doses (8).’ None of the previous studies reported the prevalence of prescribing select antipsychotics or the dosing ranges used for each agent.

In this study, nearly a third of patients were prescribed ≥ 1 antipsychotic above the recommended typical maximum dose and/or taking two or more antipsychotics. The most commonly prescribed antipsychotics were olanzapine and clozapine. At SASH, clozapine trough levels are routinely collected as clinically indicated to help guide dosing. For other antipsychotics, however, dosing is typically guided by subjective reports of tolerability/effectiveness. This may explain why the dose range for clozapine was lower compared to other antipsychotics; however, some patients may have been on a lower dose because it was in the process of being titrated. Most olanzapine orders were in the 30–40 mg daily dose range, which is cited as a non-inferior treatment option to clozapine in patients with treatment-resistant schizophrenia (TRS) (22, 23). These data suggest that there is a high prevalence of patients with TRS at SASH. SASH policy requires prescribers to obtain approval from the medical director before initiating a patient on APP, and to document in the medical record that the patient has failed more than two previous antipsychotic trials at adequate dose and duration. But because this cannot always be reliably ascertained (whether the term ‘treatment failure’ is being used appropriately/consistently among all prescribers), data on the prevalence of TRS in the current study sample was not reported.

Other antipsychotics that were used at above recommended typical maximum doses were haloperidol and fluphenazine. Although it used to be more common practice to use higher doses of first-generation antipsychotics, it has since been suggested that, on average, the maximum effective dose of haloperidol and fluphenazine may be closer to doses of 5 mg per day (24, 25). The ongoing use of high-dose first-generation antipsychotic use in this setting, as well as overall high-dose antipsychotic/APP use, underscores the need for antipsychotic stewardship to oversee and systematically report on potentially dangerous prescribing practices in this vulnerable population.

Approximately 25% of individuals with schizophrenia spectrum disorders (with or without forensic involvement) do not respond to treatment (26). Inpatient psychiatrists/advanced practice providers in state psychiatric hospitals strive to improve symptoms enough to where patients can eventually transition to a less restrictive treatment setting. As such, the perceived potential to improve symptoms enough to work towards hospital discharge may outweigh the perceived potential risks of trialing higher than recommended antipsychotic dosing strategies or APP. The problem is, it is challenging to accurately determine the effectiveness of the higher dose or APP, usually due to the subjective nature of assessing medication response, as well as inconsistent/unreliable documentation. Furthermore, empirical evidence suggests adverse events due to medication errors and toxicity are frequent, with the odds of experiencing an adverse event increasing with length-of-stay (27, 28). This further emphasizes the need for improved patient safety monitoring in state psychiatric hospitals, as this study suggests the average length of stay in this setting is typically years long.

The lack of biomarkers to guide diagnosis and treatment remains the largest barrier to improving care for people with SMI (13), in addition to the lack of effective treatment options for a significant portion of patients. These barriers culminate and contribute to the use of high antipsychotic doses/APP. Methods to measure treatment efficacy are highly subjective, even with the use of rating scales (29). Specific rating scales (e.g., Positive and Negative Syndrome Scale [PANSS], Brief Psychiatric Rating Scale [BPRS] and Clinical Global Impressions [CGI] scores) are recommended for measuring antipsychotic treatment response using standard definitions (e.g., remission, response) (30). While this is the standard method of measuring antipsychotic efficacy in clinical trials, rating scales are rarely used in clinical practice (31). There are multiple barriers to using measurement-based care tools, such as time constraints, negative attitudes/lack of clarity on the clinical utility by individual practitioners, and/or lack of training in how to use them appropriately (32). Safety outcomes such as adverse effect reporting (e.g., hyperprolactinemia, extra-pyramidal side-effects, cardiac side-effects, and metabolic disorder) are not always reliably reported (33–35). When they are reported, it is a time-intensive endeavor to evaluate the precise timeline of symptom reporting and medication changes. It will take designated workgroups and changes in health policy to incentivize creating enhanced monitoring systems that can better track efficacy and safety outcomes associated with antipsychotic prescribing patterns (18).

Establishing standardized policies and procedures for monitoring antipsychotic use patterns and associated outcomes (e.g., response/remission rates using validated rating scales, time to treatment response/remission, antipsychotic dosing patterns associated with achieving treatment response/remission and adverse effects) should be a high priority for state psychiatric hospitals. Currently, there are no established programs/procedures for collecting/analyzing these data, and retrieving the necessary data often is not feasible or readily accessible (36). For example, when an antipsychotic is discontinued, the rationale is often documented only once in a single progress note amid hundreds. Sometimes the stated reason for antipsychotic discontinuation is ineffectiveness, and other times it’s because the patient began refusing the medication. Regardless, the medication often is listed as a “previously failed medication trial,” when many factors leading to discontinuation may not necessarily constitute a true treatment ‘failure’ (e.g., treatment nonadherence, intolerability). Since a large contributor to recidivism and rehospitalization is medication nonadherence (37, 38), it is important to have standardized comprehensive medication histories that are reliable and readily accessible to all members of the patient’s healthcare team (39).

**Figure 1:**
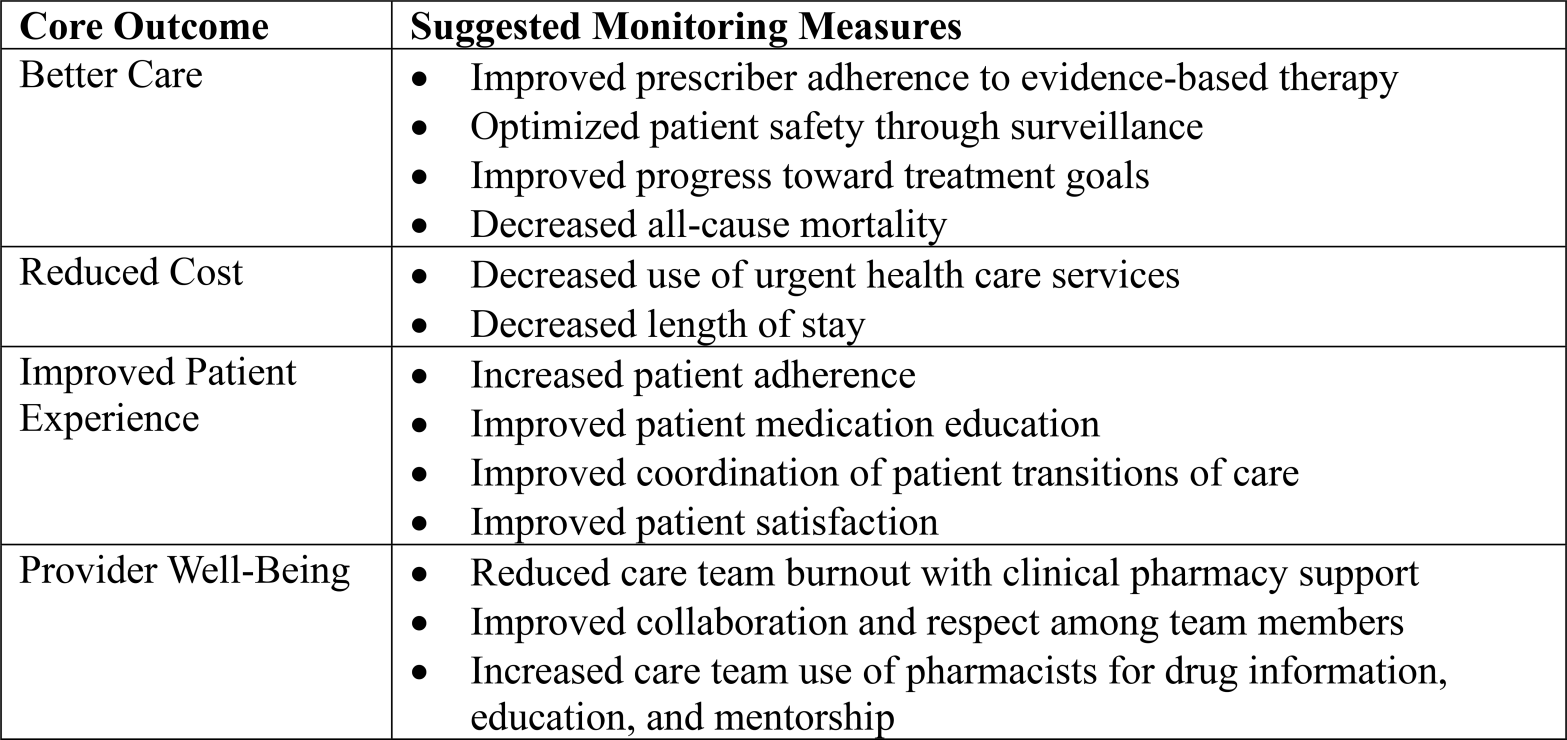
Recommended monitoring for achieving antipsychotic stewardship core outcomes.

A feasible way to begin establishing standardized antipsychotic monitoring procedures is to pilot the development and implementation of ASPs (20). Board-certified psychiatric pharmacists (BCPPs) are an underutilized resource that can address the need for increased access to cost-effective and higher-quality patient care in state psychiatric hospitals (40). The American Association of Psychiatric Pharmacists envisions that every patient with a psychiatric diagnosis will have their medication treatment plan reviewed, optimized, and managed by a psychotropic stewardship team with a BCPP co-leader. It is recommended that ASPs track the following four core outcomes (1): better care, (2) reduced cost, (3) improved patient experience, and (4) provider-well-being (41). **Figure 1** outlines suggested monitoring measures for achieving these core outcomes. Utilizing BCPPs to oversee ASP responsibilities could help ensure antipsychotic monitoring procedures are enforced while minimizing the impact on individual clinicians’ workload.

This study has limitations that should be taken into consideration. Most notably, this was a single-center, cross-sectional study, utilizing chart data from one single point in time. As such, these findings are not generalizable to all state psychiatric hospitals. Rather, these data may serve as a starting point for future studies to expand upon. Furthermore, since this was a cross-sectional study, it is not possible to know whether antipsychotics were in the process of being titrated/tapered, or whether patients were acutely ill versus at their presumed psychiatric ‘baseline.’ As stated in a previous study, a snapshot of patients in any forensic setting will skew data toward those with a longer length of stay (9). The study sample did not allow for meaningful comparisons in antipsychotic prescribing patterns between forensic and non-forensic admission types, since only a small sample of patients in the study sample were considered non-forensic admissions (n < 30). Lastly, antipsychotic serum levels were not available for most patients and were thus not collected/reported. Data pertaining to health outcomes were also not able to be feasibly collected to determine whether certain antipsychotic prescribing patterns are associated with select outcomes.

In conclusion, APP and high-dose antipsychotic use was common in one U.S. state psychiatric hospital. Larger, multicenter studies are needed to compare antipsychotic prescribing patterns between psychiatric hospitals. Since state psychiatric hospitals treat an exceptionally vulnerable patient population (patients with SMI on forensic/civil involuntary treatment commitments), community partners and policymakers are encouraged to support the development of ASPs.

## Data Availability

The datasets presented in this article are not readily available because permissions for access to the generated datasets would need to be approved by the Texas State Health and Human Services Commission. Requests to access the datasets should be directed to vadiei@uthscsa.edu.
